# Customized biofilm device for antibiofilm and antibacterial screening of newly developed nanostructured silver and zinc coatings

**DOI:** 10.1186/s13036-023-00326-y

**Published:** 2023-03-06

**Authors:** Daniele Ghezzi, Marco Boi, Enrico Sassoni, Francesco Valle, Elena Giusto, Elisa Boanini, Nicola Baldini, Martina Cappelletti, Gabriela Graziani

**Affiliations:** 1 Biomedical Science and Technologies and Nanobiotechnology Lab, IRCCS Istituto Ortopedico Rizzoli, Via Di Barbiano 1/10, 40136 Bologna, Italy; 2 Department of Civil, University of Bologna, Chemical, Environmental and Materials Engineering, Via Terracini 28, 40131 Bologna, Italy; 3 Institute of Nanostructured Materials, National Research Council (ISMN-CNR), Via Piero Gobetti, 101, 40129 Bologna, Italy; 4 Blizard Institute, Queen Mary University of London, 4 Newark St, London, E1 2AT UK; 5 Department of Chemistry, University of Bologna, Giacomo Ciamician”, Via Selmi 2, Bologna, Italy; 6 Department of Biomedical and Neuromotor Sciences, University of Bologna, Via Massarenti 9, 40128 Bologna, Italy; 7 Department of Pharmacy and Biotechnology, University of Bologna, Via Irnerio 42, 40126 Bologna, Italy

**Keywords:** Ionized Jet Deposition, Calgary Biofilm Device, Antibacterial, Antibiofilm, Metal coatings, Biocompatibility

## Abstract

**Background:**

Bacterial colonisation on implantable device surfaces is estimated to cause more than half of healthcare-associated infections. The application of inorganic coatings onto implantable devices limits/prevents microbial contaminations. However, reliable and high-throughput deposition technologies and experimental trials of metal coatings for biomedical applications are missing. Here, we propose the combination of the Ionized Jet Deposition (IJD) technology for metal-coating application, with the Calgary Biofilm Device (CBD) for high-throughput antibacterial and antibiofilm screening, to develop and screen novel metal-based coatings.

**Results:**

The films are composed of nanosized spherical aggregates of metallic silver or zinc oxide with a homogeneous and highly rough surface topography. The antibacterial and antibiofilm activity of the coatings is related with the Gram staining, being Ag and Zn coatings more effective against gram-negative and gram-positive bacteria, respectively. The antibacterial/antibiofilm effect is proportional to the amount of metal deposited that influences the amount of metal ions released. The roughness also impacts the activity, mostly for Zn coatings. Antibiofilm properties are stronger on biofilms developing on the coating than on biofilms formed on uncoated substrates. This suggests a higher antibiofilm effect arising from the direct contact bacteria-coating than that associated with the metal ions release. Proof-of-concept of application to titanium alloys, representative of orthopaedic prostheses, confirmed the antibiofilm results, validating the approach. In addition, MTT tests show that the coatings are non-cytotoxic and ICP demonstrates that they have suitable release duration (> 7 days), suggesting the applicability of these new generation metal-based coatings for the functionalization of biomedical devices.

**Conclusions:**

The combination of the Calgary Biofilm Device with the Ionized Jet Deposition technology proved to be an innovative and powerful tool that allows to monitor both the metal ions release and the surface topography of the films, which makes it suitable for the study of the antibacterial and antibiofilm activity of nanostructured materials. The results obtained with the CBD were validated with coatings on titanium alloys and extended by also considering the anti-adhesion properties and biocompatibility. In view of upcoming application in orthopaedics, these evaluations would be useful for the development of materials with pleiotropic antimicrobial mechanisms.

**Graphical Abstract:**

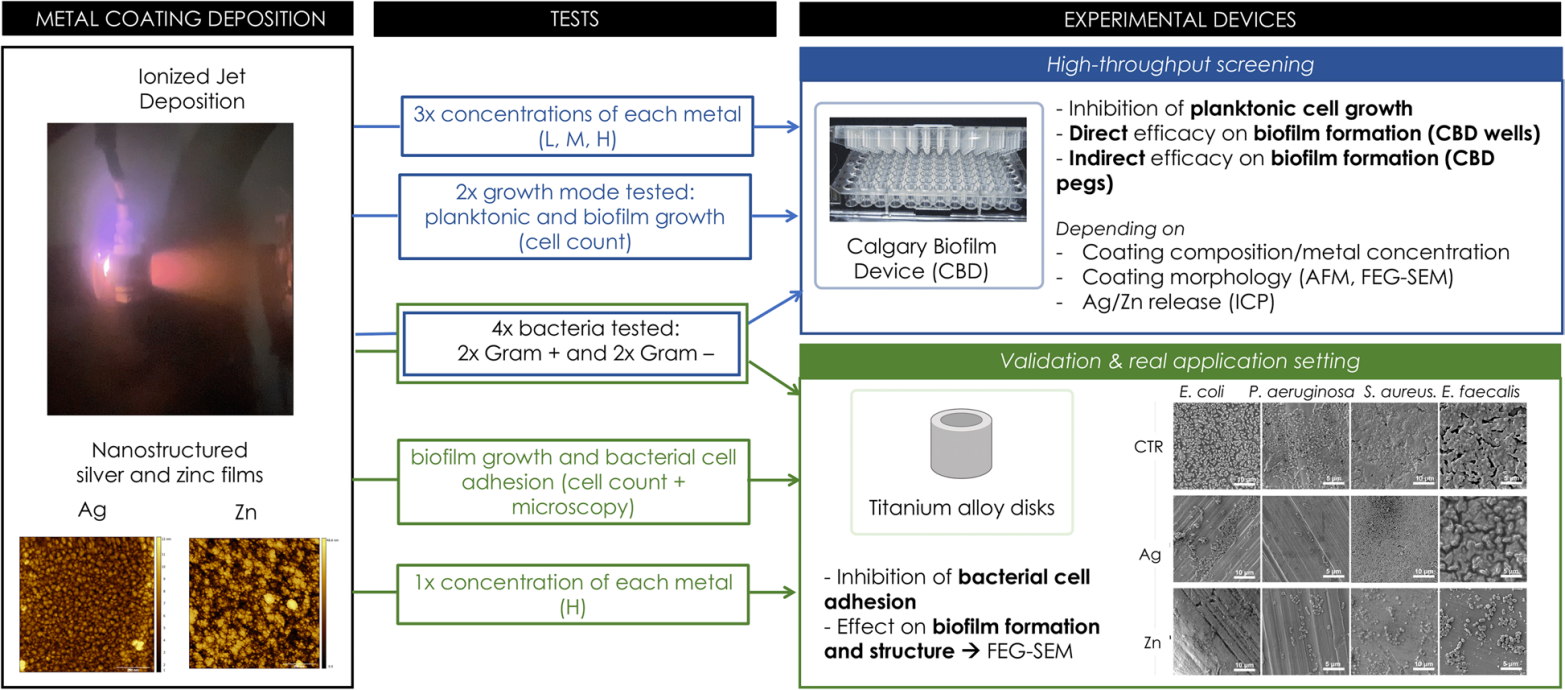

**Supplementary Information:**

The online version contains supplementary material available at 10.1186/s13036-023-00326-y.

## Background

Bacterial infections are among the most severe complications mostly caused by the contamination of implantable and non-implantable biomedical devices and surfaces, including heart valves, endovascular stents, joint prostheses, implantable meshes, artificial lenses, cochlear implants, surgery room floors, anesthesia carts, operating tables, and surgery tools [1–3].

Surgical infections are frequent (incidence rate up to 20% depending on surgery type and comorbidities [4]), resulting in ~ 4 million cases per year in Europe [5]. These infections pose an important societal and economic burden directly causing about 37.000 deaths/year and resulting in a high direct cost of ~ 7 billion Euros, accounting for surgery, therapy, and prolonged hospitalization [5].

In orthopaedics, the treatment of infection is challenging, as it often requires the removal of the orthopaedic implant, the debridement of the tissues, and revision surgery; therefore, prevention measures are preferred [6].

To address infection, the gold standard relies on systemic antibiotic therapy, which requires high doses of antibiotics and maximizes the risk of systemic toxicity and side effects, promoting the development of resistant bacterial strains [6, 7]. Controlled antibiotic release systems such as antibiotic impregnated devices or antibiotic loaded coatings are also used, as they allow the delivery of higher concentrations of antibiotics directly into the infection site, thus enhancing efficacy and lowering systemic toxicity. However, these solution does not impede the development of bacterial resistances to antibiotics, which makes them increasingly ineffective [7].

To reduce the development of resistant bacterial strains, inorganic multi-spectral (and mainly metallic) antibacterial coatings are largely investigated [6]. The importance of these solutions is growing due to the progressive spread of custom-made 3D printed protheses and of surface patterning, resulting in highly porous and highly rough implants, which tend to favour bacterial adhesion, biofilm formation, and infection [8, 9].

However, bacterial contaminations leading to infections do not only derive from the implanted prosthesis but also from the operators, their Personal Protective Equipment (PPE), the surgical equipment, the surface in the operating theatre, the common rooms, the patient stay in the hospital, or even at home after the intervention [2, 3]. For this reason, the use of antibacterial surfaces and PPE is raising increasing interest.

The microbial biofilm is one of the main issues causing infections from contaminated orthopaedic prostheses [10]. A biofilm is composed of a structured community of microbial cells that are firmly attached to a surface and have unique metabolic and physiological attributes. The biofilm formation is the result of sequential and coordinated steps that involve a first initial bacterial adhesion on the surface and then the maturation of the biofilm that includes the secretion of a matrix composed of a complex array of extracellular polymeric substances (EPS) [10–12]. The extracellular matrix plays key roles in hampering the penetration of harmful molecules and protecting the microbial community from physical and chemical damages (*i.e.*, dehydration, UV radiation, detergents, temperature shifts) [10]. Therefore, biofilm growth conditions provide specific advantages compared to the planktonic growth in terms of resistance to environmental stress and toxic compounds like antibiotics and metals [11]. Under biofilm growth conditions, the high proximity between bacterial cells also promotes both horizontal gene transfer and intercellular communication (quorum sensing) that might prompt the transmission of antibiotic resistance genes and the possible onset of multidrug resistant microbes [12].

The cell wall is the first barrier that an antimicrobial compound, like a metal ion, must overcome. Based on their envelope composition, bacteria can be categorized as gram negative or gram positive, depending on the nature of the cell envelope [13]. Bacteria from both these groups can form biofilms on different organic and inorganic surfaces. Most of the infection-related biofilm formation is caused by gram-positive staphylococci and enterococci strains, which provoke severe outcomes [14]. Among these, *Staphylococcus aureus* is the most common microbial species responsible for prosthetic joint infections and includes several virulent strains with high capabilities to form hard-to-erase biofilms [15]. *Enterococcus faecalis* is one of the major pathogens in nosocomial (hospital-acquired) infections, which often occur via medical devices [16]. Although with a minor incidence, also gram-negative species have been detected in prosthetic joint infections including *Escherichia coli* and *Pseudomonas aeruginosa* [14, 17]. In particular, the latter is a strong EPS-producer being able to quickly form robust biofilms on abiotic and biotic (*e.g.*, human tissues) surfaces [18].

Metals are used as antimicrobials due to both their efficacy at low dosage and the complexity for microbes to develop metal resistances [19]. To address infections, the use of metal-based inorganic coatings and nanoparticles is promising, as they have toxic effects against multiple molecular targets within the microbial cell [12, 20]. Silver coatings have been widely investigated and used in the clinical practice. However, traditional coatings exhibit some drawbacks, including insufficient adhesion to the substrate, scarce uniformity, and scarce control over silver ions release. In addition, when applied to prostheses, due to their micrometric thickness, they tend to alter the implant morphology at the micro-scale level (*i.e.*, porosity and surface finishing), which is designed to maximize the osseointegration. Finally, bacterial interactions with biomaterials can be different for planktonic (suspended) and biofilm cells, hence the properties of the coatings need to be addressed by considering both antibacterial and antibiofilm activities [21]. Despite the promising features of metal-based coatings to functionalize biomedical devices and surfaces, only a few studies have systematically investigated both antibacterial and antibiofilm activities against different bacterial strains. Furthermore, the methodologies aiming at exploring the efficacy of promising antimicrobial coatings are usually based on standard microbiological tests, including top agar assays and inhibition of the growth in liquid cultures, with the limitation of the low number of simultaneous tests [6, 7]. The typical outcomes of these tests are the determination of the Minimal Inhibitory Concentration (MIC) and the Minimal Bactericidal Concentration (MBC) of the antimicrobial agent [22]. For this purpose, the use of high-throughput technologies represents a key step for performing advanced experiments with high reliability and allowing the simultaneous investigation of multiple experimental conditions, including biofilm formation assays.

In this work, the Calgary Biofilm Device (CBD) was used, for the first time, as high-throughput device to explore the possibility to develop and use new generation nanostructured silver- and zinc-based films as coatings for biomedical devices with antibacterial and antibiofilm properties. The CBD device consists of a 96-well microtiter plate and a coverlid presenting 96 supports (named pegs) that perfectly fit into the wells when the plate is closed [23–25]. The pegs are used as supports for biofilm formation as they are partly submerged in the liquid medium inoculated with the bacterial strain in the wells. The wells of the CBD were used as deposition substrate for metal-based coatings that were applied by using a novel plasma-assisted technique named Ionized Jet Deposition (IJD). This is a deposition technology that preserves the stoichiometry from the target to the coatings even in the case of complex materials and provides both a nanostructured and high specific surface to guarantee a sustained release of the antibacterial agent [26–29]. After deposition on the CBD, the composition and morphology of silver and zinc coatings, as well as their ion release, were characterized together with the antibacterial properties and their capability to inhibit the biofilm formation by four model human bacterial pathogens, two gram-positive and two gram-negative strains*.* Finally, the coatings were applied and tested on a microstructured titanium alloy substrate to validate the results obtained with the CBD and to evaluate the effect on the bacterial adhesion (the first stage of biofilm formation).

## Results

### Physicochemical properties of silver and zinc coatings

**Fig. 1:**
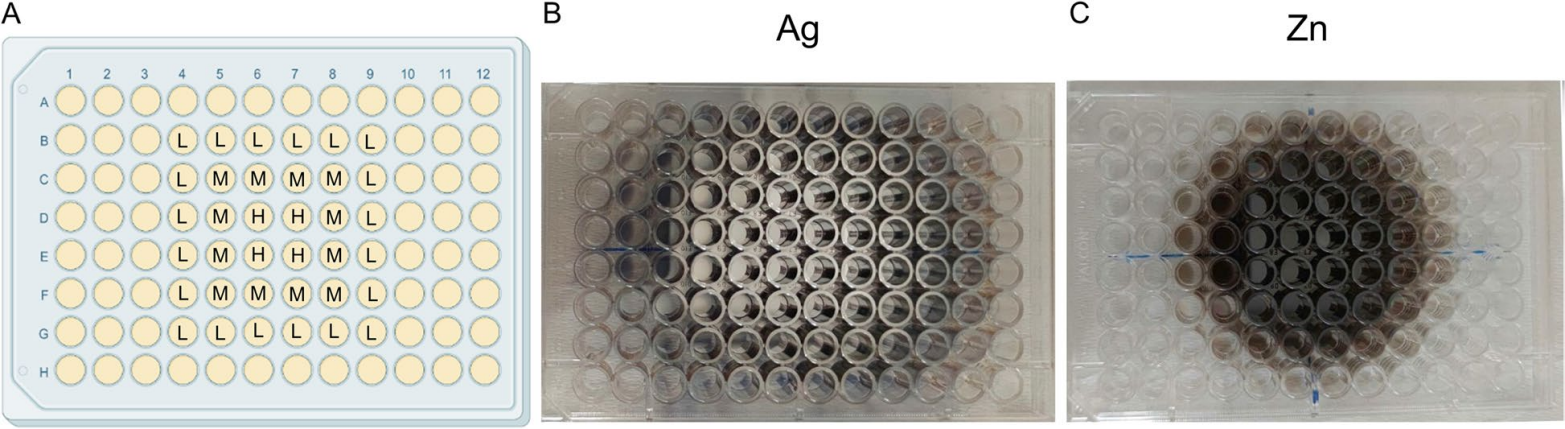
Graphical representation of the three areas (**A**) on the Calgary Biofilm Device covered with silver (**B**) and zinc (**C**) after IJD deposition. The wells depicted as L, M, and H correspond to the CBD areas presenting low, medium, and high concentration of metal coating, respectively

The coatings were obtained in one-step without pre- or post-deposition treatments by fixing the CBD plate in front of the metal target in a central position. In this manner, the support (the CBD plate) was covered by the targeted metal in a concentric way, presenting the highest concentration of the metal in the four central wells/pegs. By moving from the center towards the perimeter of the support, the metal concentration decreased, allowing the simultaneous deposition of different metal amounts on the CBD wells. Based on this, three main areas were identified in the CBD plate that were characterized by different concentrations of deposited metal, *i.e.*, low metal amount (named L) in the external area, medium metal amount (M) in the central area, and high metal amount (H) in the inner area of the CBD (Fig. 1). Ideally, if a biomedical device is placed in the same positions during the deposition, the same coating, composition, thickness, and characteristics can be reproduced.

**Fig. 2:**
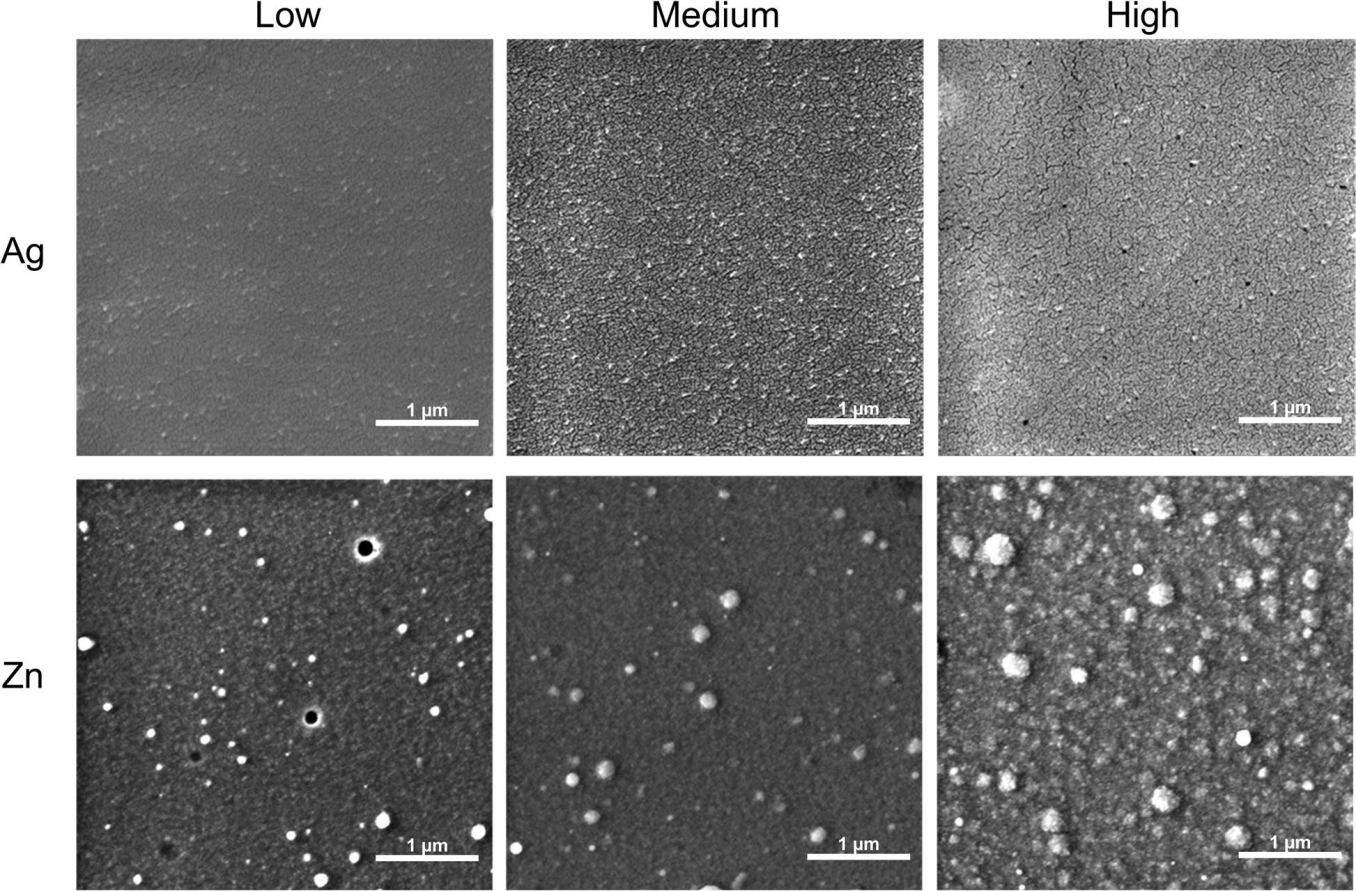
FEG-SEM images of Ag and Zn coatings deposited at the three different concentrations tested (low (L), medium (M), and high (H))

Morphology of the coatings, as assessed by Field Emission Gun Scanning Electron Microscopy (FEG-SEM), is reported in Fig. 2. Both Ag and Zn films showed a nanostructured morphology and are composed by nanosized aggregates, although relevant differences were observed (Fig. 1 and Table 1). Silver films were composed by spherical aggregates, creating a very homogeneous surface morphology, while zinc coatings were constituted by aggregates that were generally coarser and less regular in shape. In particular, a significant coarsening of the zinc aggregates was visible in zinc coatings deposited in the inner area of the CBD (area H in Fig. 1).

**Table 1:** Diameters of Ag and Zn aggregates on the coatings. Sparse aggregates of larger dimensions are neglected

	Minimum diameters (nm)	Maximum diameters (nm)
Ag-H	6	47
Ag-M	14	49
Ag-L	10	33
Zn-H	7	116
Zn-M	7	100
Zn-L	7	103

**Fig. 3:**
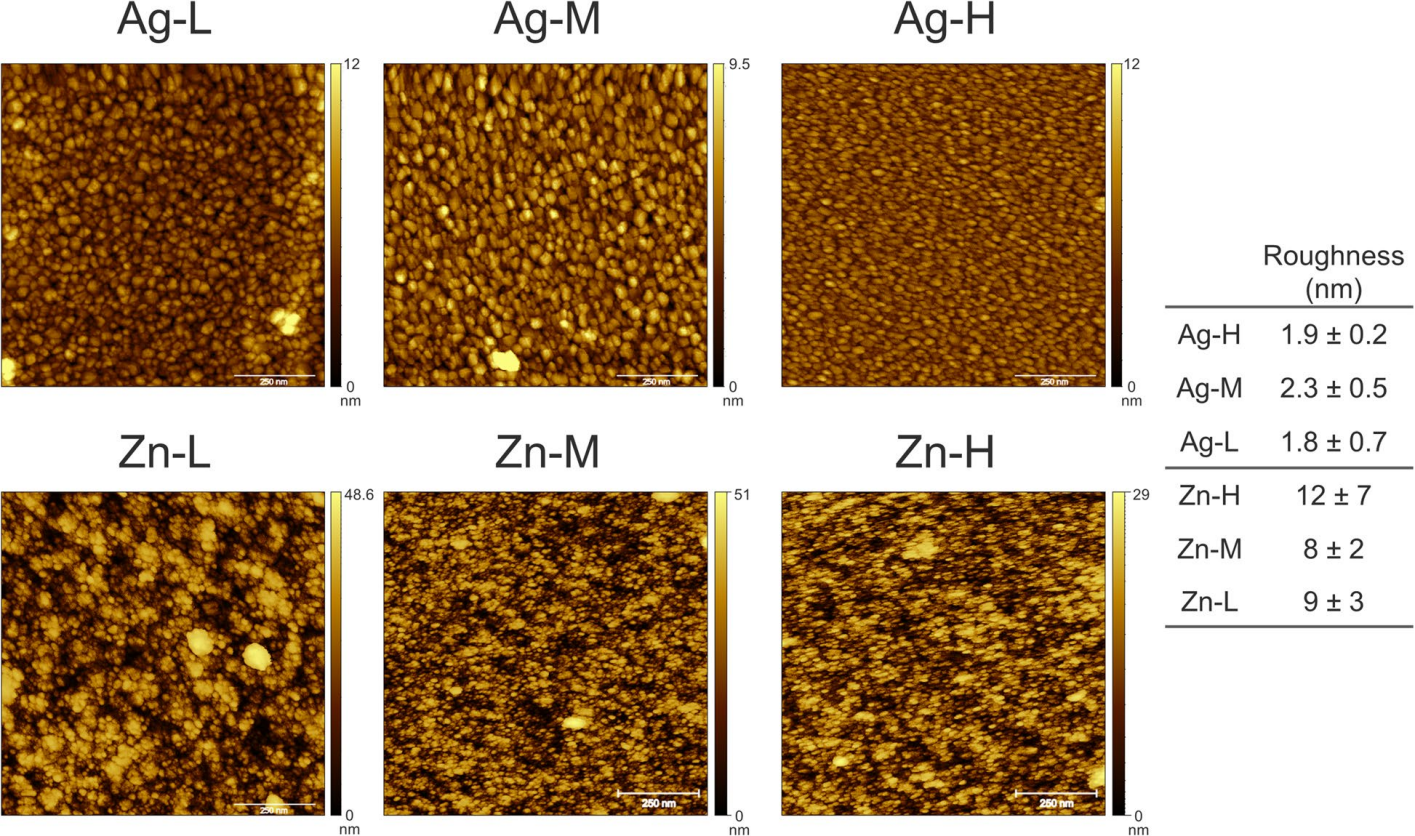
AFM images and roughness values of Ag and Zn coatings deposited at the low (L), medium (M), and high (H) concentrations. The scalebar is set to 250 nm

Surface topography, as assessed by Atomic Force Microscope (AFM), is shown in Fig. 3. Silver coatings were composed by aggregates whose dimensions ranged from about 5 to 50 nm, with the maximum and minimum diameters depending on the position with respect to the plasma plume (Table 1). Maximum diameters of aggregates featuring silver coating, observed but not included in Table 1 (40 nm, 91 nm, and 52 nm in L, M, and H, respectively), are influenced by the presence of coarse clusters, randomly distributed on the surface. If these aggregates are neglected, a general coarsening is noticed in the central area and inner area, with the aggregates progressively increasing in diameter, from 33 nm (L) to about 50 nm (M and H), as also seen in the case of increasing deposition times. Important differences were also present in the shapes of the aggregates in the different areas; indeed, those in the external areas showed a more elliptical shape, due to a different angle of incidence between the plasma plume and the surface.

Surface roughness of silver coatings is shown in Fig. 3. Values of root mean square (RMS) of 1.9 ± 0.2, 2.3 ± 0.5, and 1.8 ± 0.7 nm are observed in the inner (H), central (M), and external (L) area, respectively. Therefore, sample roughness was essentially the same for all silver films, regardless of the position. On the other hand, the heterogeneity of surface roughness increased by moving from the inner (H) to the external (L) area of the CBD.

For all zinc coatings, the minimum diameter was 7 nm, while the maximum was slightly higher (≈115 nm in the inner (H) area), and lower (≈ 100 nm) in the external (L) one. Again, the presence of isolated larger aggregates (diameters 85–115 nm) could be noticed in the inner (H) and central (M) areas (Fig. 3). As for silver, in M and L the distribution of aggregates of larger size was random and defined the highest diameter detected. In the case of zinc, although the dimension of the single aggregates did not depend on the position of the well, larger clusters could be observed in the central (M) and in the inner (H) areas, as already noticed by SEM. The external (L) samples showed a more elliptical shape of the aggregates, consistently with the different incidence angle.

With zinc, the roughness was higher for the inner (H) area, while differences among the other areas were negligible. This correlates with the higher amount of large size clusters, which also causes higher heterogeneity. Roughness of zinc films was about ten-fold higher than that of the silver films, probably because of a more scattered distribution of the aggregates shape. At the same time, the heterogeneity was also higher, because of the more irregular shape and dimension distribution of zinc clusters compared to silver ones.

The analysis of coating thickness revealed that the silver layer was rather uniform. A 20–22 nm decrease from the inner to the outer position was observed, with the H area showing 63 nm of thickness. The zinc layer was thicker and less uniform compared to silver, with L and M areas presenting thickness of 150 and 270 nm, respectively. The zinc layer corresponding to the H area was too hard to scratch, therefore its thickness was not determined.

**Fig. 4:**
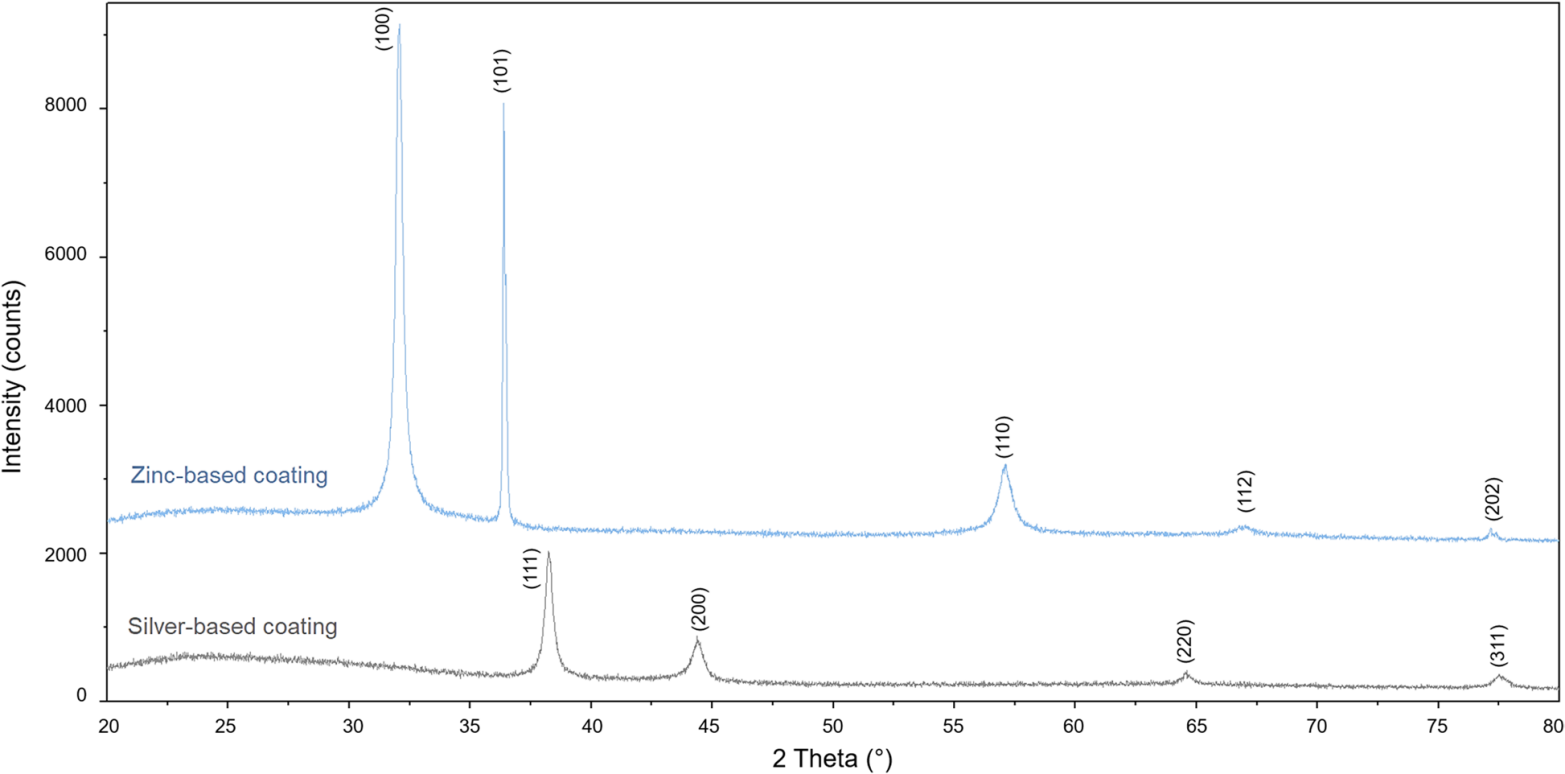
XRD spectra of Ag and Zn films. All peaks in the Ag coating are attributed to metallic silver (ICDD 00–004-0783), while those in the Zn coating are attributed to zinc oxide (ICDD 00–036-1451). The Miller Indices (hkl) corresponding to each peak are reported

Silver and zinc-based films composition, as assessed by X-Ray Diffraction (XRD), is shown in Fig. 4, where the Miller Indices (hkl) corresponding to each diffraction peak are reported. Ag coatings were composed of metallic silver (ICDD 00–004-0783), while no signs of formation of oxides, nitrates, or other corrosion phases were present, according to previous results of the research group [30]. Zinc films were composed of zinc oxide (ZnO, ICDD 00–036-1451), which is consistent with deposition in oxygen atmosphere. The presence of peaks at 32.02°2θ (100 reflection) and 36.04°2θ (101 reflection) and the absence of the peak at 34.4°2θ (002 reflection), suggest a preferential orientation of the ZnO crystals, as also found elsewhere for copper-based nanostructured thin films [31].

Silver concentration in the wells was measured by Inductively Coupled Plasma (ICP) and is shown in Table 2. Silver concentration was the same in the inner and central area, consistently with their surface roughness. Instead, it was lower and more variable in the external area. With zinc, the inner and central area showed the same roughness but not the same release, probably because of the coarsening of grains in the central area. By comparing zinc and silver, the concentration of the first in the well was significantly higher. This is probably related with the higher surface roughness.

**Table 2:** Amount of ions released from Ag and Zn coatings during 48 h of incubation

	Ion release (ppm)
Ag-H	2.03 ± 0.06
Ag-M	2.03 ± 0.14
Ag-L	1.79 ± 0.26
Zn-H	291 ± 39
Zn-M	228 ± 43
Zn-L	162 ± 42

### Antibacterial and antibiofilm activities of the Ag and Zn coatings deposited on the Calgary Biofilm Device system

The coatings deposited on the CBD wells were tested for the antibacterial and antibiofilm properties by considering the inhibition of the bacterial growth as both planktonic and biofilm cells of four model human pathogens, two gram-negative strains of *Escherichia coli* and *Pseudomonas aeruginosa* species and two gram-positive strains of *Staphylococcus aureus* and *Enterococcus faecalis* species. The structural characteristic of the CBD allowed to test in parallel the coatings effect on the planktonic cells growing in the medium in each well, the biofilm cells growing on the coated wells, and the biofilm cells growing on the uncoated pegs.

**Fig. 5:**
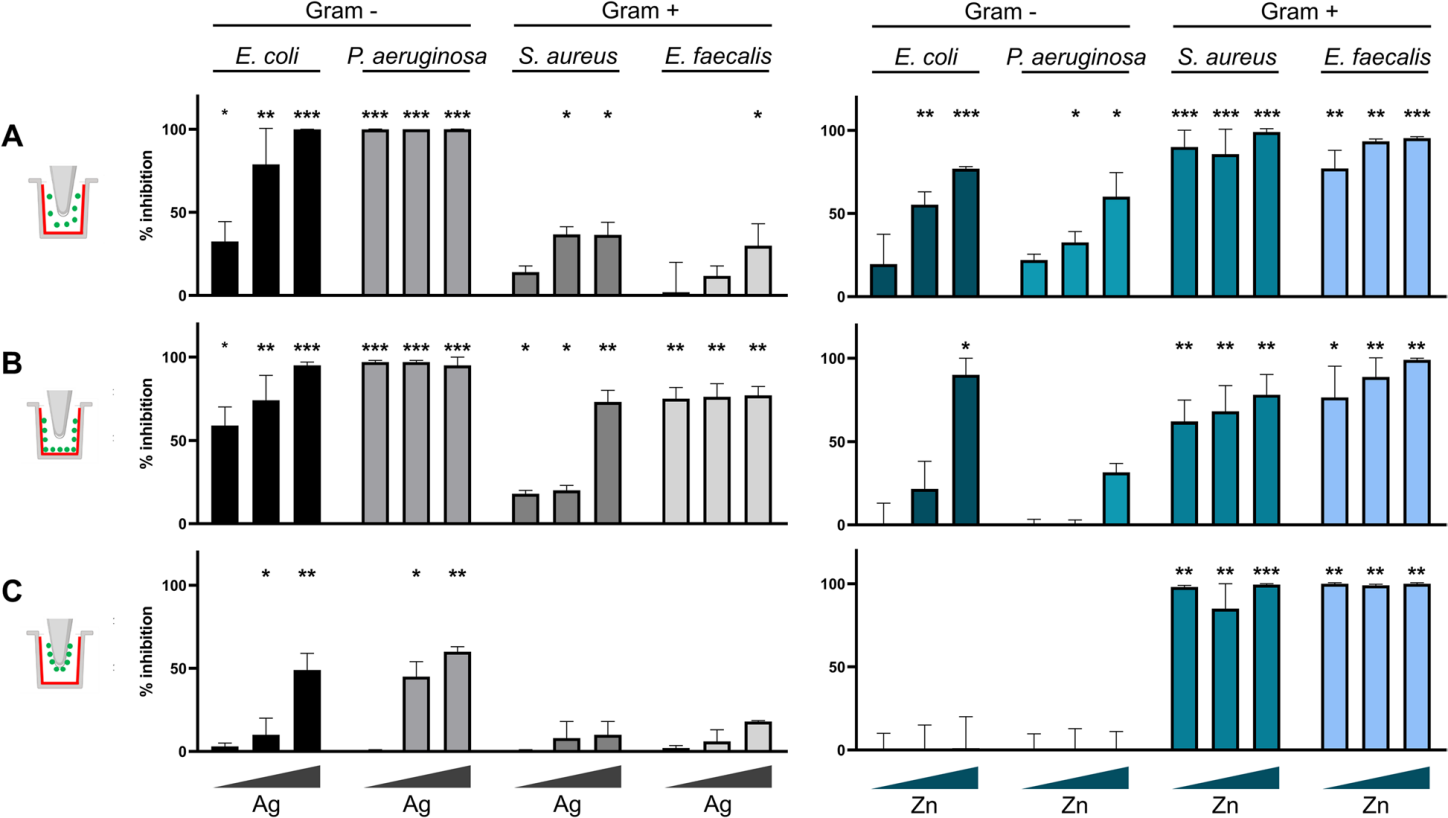
Antibacterial and antibiofilm activities exerted by Ag (left) and Zn (right) coatings against the four bacterial pathogens *E. coli*, *P. aeruginosa*, *S. aureus*, and *E. faecalis*. The CBD icon on the very left represents the type of analysis corresponding to each panel, with the coating in red and the measured bacterial planktonic cells/biofilm in green. The results are reported as the mean of the percentage of bacterial and biofilm inhibition ± standard deviation. The triangles below each histogram indicate the increasing concentration of each metal tested (from low (L), through medium (M), to high (H)). Statistical analysis was performed using one-way Anova test. Differences were considered significant compared to control (bacterial growth w/o coating) for *p* < 0.05 (* *p* < 0.05, ** *p* < 0.01, *** *p* < 0.001). **A** Inhibition of the bacterial planktonic growth. **B** Inhibition of bacterial biofilm formation on the coated well. **C** Inhibition of the bacterial biofilm formation on the non-coated peg. The absorbance values (OD_595_) of crystal violet (CV) associated with the amount of biofilm on wells and pegs without metal coating (the control experiment) are reported in Table S1

In this study, the Ag coatings showed higher activity against the gram-negative *E. coli* and *P. aeruginosa* strains compared to the gram-positive strains. Indeed, the planktonic cells of *E. coli* and *P. aeruginosa* were significantly inhibited by the Ag coatings even at the minimum silver concentration tested, with *P. aeruginosa* showing 100% of inhibition in the presence of all the three concentrations. On the other hand, the planktonic cells of the gram-positive bacteria *S. aureus* and *E. faecalis* exhibited a significant sensitivity to silver in two out of the three concentrations tested, showing more than 80% of growth inhibition in the wells in the H area. In regard with the biofilm formation, the percentage of the biofilm inhibition on the coated wells was significant for all the bacterial strains, although the percentage of inhibition greatly differed among the strains. Specifically, the percentage of biofilm inhibition was 100% for *P. aeruginosa* in the presence of all the three silver concentrations under analysis, whereas *S. aureus* demonstrated higher resistance to the Zn coatings in L and M areas, with around 20% of biofilm formation inhibition. The antibiofilm activity of the Ag coatings against *E. faecalis* ranged between 70% and 80% under all the tested concentrations, while for *E. coli* the inhibition varied from 60% in L to almost 100% in H. On the other hand, the effect of Ag coating on the biofilm formation on the uncoated pegs was significant only for *E. coli* and *P. aeruginosa* showing a maximum percentage of inhibition of 50% and 60%, respectively (Fig. 5).

The gram-positive *S. aureus* and *E. faecalis* strains generally resulted more sensitive to the Zn coating on the CBD as respect to the gram-negative strains in terms of both planktonic growth and biofilm formation. Zinc-coated wells provoked a significant decrease in the planktonic cell growth for all the four pathogens, approaching 100% of inhibition with *S. aureus* under all the tested conditions and with *E. coli* and *E. faecalis* at the highest metal concentration tested. In consideration of the biofilm formation on zinc-coated wells, gram-negative strains *E. coli* and *P. aeruginosa* did not show any significant reduction, while this was highly reduced for *S. aureus* and *E. faecalis* in the presence of all the metal concentrations tested. A similar trend was also observed by evaluating the effect of zinc-coated wells on the formation of biofilm on the uncoated pegs, with no reduction on gram-negative strains and almost total inhibition for the two gram positive (Fig. 5).

### Proof-of-concept of application for implantable devices

#### Antibiofilm activity of Ag and Zn coatings on titanium alloys

Silver and zinc deposited on titanium alloys were tested for their capacity to prevent the bacterial biofilm formation and bacterial adhesion. In general, the behaviour of the biofilm growth and adhesion on coated alloys was in line with the results obtained from the CBD experiments. The Ag-coated titanium alloys were highly toxic for *P. aeruginosa* and *E. coli* reducing the bacterial adhesion of more than 70% compared to the non-coated alloys. Zn-coated titanium alloys were highly effective against the adhesion of the gram-positive *E. faecalis*, whereas the anti-adhesion of *E. coli* was the least affected (Table 3).

**Table 3:** Inhibition of bacterial cells adhesion on silver- and zinc-coated titanium alloys

	Ag coating (%)	Zn coating (%)
*E. coli*	69.3 ± 1.2	14.6 ± 0.3
*P. aeruginosa*	71.8 ± 2.0	49.3 ± 4.1
*S. aureus*	39.6 ± 1.8	42.7 ± 5.1
*E. faecalis*	17.5 ± 1.2	80.0 ± 0.4

**Fig. 6:**
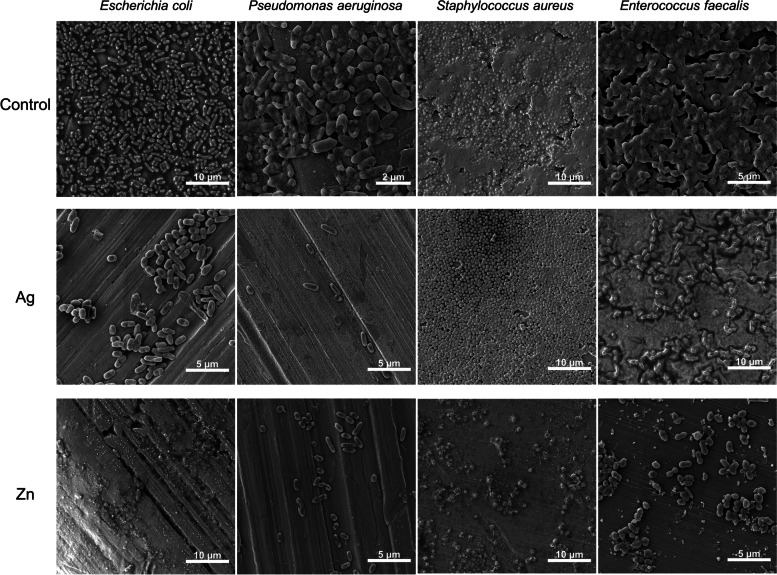
SEM images of the titanium alloys without coating (CTR), and with Ag and Zn coatings after 48 h incubation with bacterial cultures of *E. coli*, *P. aeruginosa*, *S. aureus*, and *E. faecalis*

SEM images of the bacterial biofilms formed on the coated side of the titanium alloy showed the preservation of *S. aureus* and *E. faecalis* cell morphologies on the Ag coating. While the cells of *S. aureus* did not seem to be embedded in the extracellular matrix that was visible in the control samples (non-coated alloys), the main structure of the *E. faecalis* biofilm was maintained. On the other hand, the number of adherent cells of *E. coli* and *P. aeruginosa* was strongly reduced on Ag coatings when compared to the control samples, in which a multi-layered biofilm of these strains was present. Further, the cells of gram-negative *E. coli* and *P. aeruginosa* also showed cracks and alterations. SEM images of biofilms on Zn coatings mostly showed the adhesion of a few number or small aggregates of cells for all the strains tested. Some extracellular matrix was visible in the case of the only *E. coli* and *S. aureus* strains (Fig. 6).

#### Ion release

Ag and Zn release at different timepoints are in Fig. S1. Results showed that release of both metals progressively increased up to 7 days, when a sustained release was still measured. No significant differences were noticed in the release profile of the two metals except for the differences in the extent of release of the two metals, being higher for zinc.

#### Effect of Ag and Zn coatings on L929 cells

For the longest timepoint of release (7 days), MTT on coated cylinders showed that both Ag and Zn coatings are not cytotoxic (cells viability > 75%, ISO-10993–5-2009). For silver, viability was higher compared to the control (Table 4), indicating complete absence of toxicity and a slight positive effect on L929 cells viability, as also found in [28]. For zinc, slight reduction was found in cells viability, although it is still to be considered acceptable for implantable devices.

**Table 4:** Cytotoxicity of Ag and Zn coatings, as described by MTT test. Viability is expressed as % with respect to control

Cell line	% viable cells	
	Ag coating	Zn coating
L929	121 ± 4	81 ± 2

## Discussion

Ionized Jet Deposition is a suitable technology for manufacturing nanostructured thin films with submicrometric thickness and sustained ion release [26, 27, 29–31]. Preliminary analyses on the antimicrobial activities of silver-based coatings obtained through IJD were previously provided for electrospun patches [28], giving first indications on the potential of IJD to realize antimicrobial coatings for medical applications. In this work, we characterized in detail metal-based coatings obtained with this technology by analysing their physical–chemical characteristics along with the antibacterial, antibiofilm, and anti-adhesion properties. Besides the purpose of producing suitable antibacterial metal coatings applicable both to clinic and non-clinic fields, in this work we also demonstrated the use of CBD as the support of these coating deposition to perform reliable and high-throughput antibacterial/antibiofilm tests of metal coatings. Indeed, by applying the IJD technology on the CBD, we managed to obtain multiple coated wells with the same metal concentration (and morphology per plate) and wells presenting different metal concentration ranges (in the three CBD areas low, medium, and high). Furthermore, since in CBD the biofilm can form on both the coated-well and on the uncoated peg, we could evaluate in parallel the antibacterial and antibiofilm properties of the coatings associated with the toxicity of soluble metal release (leading to bacterial decrease on the uncoated peg) and with the direct cell contact with the deposited metal aggregates (leading to bacterial decrease on the coated well).

The physical–chemical characterization of the silver and zinc films deposited through IJD demonstrated that these coating are composed of metallic silver and of zinc oxide, respectively, featured by aggregates with maximum diameters varying in the three CBD areas. However, the size increase of the aggregates was not linear with the three different CBD areas, as the medium area presented higher diameters compared to the high area. Important differences were also observed in the aggregate shape, as those in the external (low) areas showed a more elliptical shape. As already noticed for both flat and rough surfaces [28, 31], the silver films showed a very regular surface with only a few numbers of isolated surface irregularities (larger aggregates). The increase in the diameter of aggregates was previously associated with longer deposition times [31]. In our case, as the deposition time was the same for each CBD area, both the difference in the shape of aggregates and the distribution of the maximum diameters of the silver aggregates depend on a different angle of incidence of the plasma plume on the surface and on the surface shape. Indeed, the angle of incidence is the parameter that changes most between the different areas and the shape of the surface that gets into contact of the plume defines the accessibility of the coating deposition. Therefore, the glancing angle of the IJD and the shape of the substrates where the deposition occurs represent aspects to focus on for possible future upgrades of the technique, as seen for the Pulsed Laser Deposition (PLD) [32]. Differently from Ag coatings, the position of the ZnO coatings in the CBD did not correlate with the dimensions of the single grains. Moreover, both the roughness in the central wells and the heterogeneity were greater than those detected with silver coatings, due to more irregular shape and dimension distribution of the zinc oxide clusters. These differences in terms of physical–chemical characteristics of metal aggregates and coatings are probably dependent on the fact that silver films grow by juxtaposition of spherical grains, which grow onto each other until saturation of the surface and then deposit layer by layer, without changing their dimensions [31]. Instead, our results suggest that the zinc oxide aggregates tend to agglomerate in irregular clusters. As the zinc deposition proceeds, the number of clusters increases, creating a progressively more irregular and inhomogeneous film surface. This characteristic could be, in turn, a consequence of the distinct thermal conductivity, decomposition temperature, and density of the two metals that determine their interactions with the electron beam and the behavior during deposition. Indeed, when the plasma plume interacts with a metal possessing high thermal conductivity, decomposition temperature, and density, such as silver, it diffuses into a smaller volume in the bulk of the metallic target, reducing the volume expansion and the tendency to eject particles from the target. This leads to Ag coatings characterized by greater surface uniformity and a scarce presence of defects. Instead, with zinc, the emission of particles from the target, together with ions, is significant, so the morphology and topography of the films are irregular and more alike those obtained by the deposition of ceramics. These different morphologies and homogeneity of the coatings, together with the different composition, can be related with the different metal ion release (detected through ICP) in the cultural medium inside the CBD well. In the case of the Zn coatings obtained in this study, the number of ions released was higher than the silver ones and increased following the areas L, M, and H. Conversely, the silver ion release in the H and M areas was the same although the release from the coatings in the M area was more heterogeneous than in the H area.

The physical–chemical features of the IJD-produced metal coatings could be used to interpret the results we obtained from the antibacterial tests we performed, by considering both the quantity of ions released and the coating roughness/morphology. Indeed, it is known that the antimicrobial properties are related with the number of ions released by the coatings that interacts with the bacterial cells exerting toxic effects through cellular contact and metal ion internalization [33, 34]. Our results showed that the antibacterial activity of the Ag and Zn coatings towards the planktonic and biofilm cells was mostly in line with the number of ions released by the coatings in the three different CBD areas. A few exceptions regarded the results we obtained with the Ag coatings in M and H areas as in some cases planktonic and/or biofilm cells were differently inhibited in these two areas despite the same number of released ions we detected. At least in the case of biofilm, these results could be justified by considering the possible effect of the higher roughness that we detected for the Ag coating in the M area as compared to the H area. These results are in line with previous studies that reported that an increased roughness of the metal surfaces enhanced the antimicrobial performance against the bacterial adhesion and the early stages of biofilm formation of *P. aeruginosa* and *S. aureus* strains [35, 36].

The antibacterial properties of IJD-produced metal coatings on the CBD were tested on four strains representative of the most common human bacteria causing biofilm-related infections [37, 38]. The results we obtained on the antibacterial and antibiofilm activity of the two IJD-based metal coatings indicated a correlation with the Gram staining groups, *i.e.*, *E. coli* and *P. aeruginosa* as gram-negative and *S. aureus* and *E. faecalis* as gram-positive strains. In this regard, Ag coatings showed a higher toxic activity against the gram-negative bacterial strains, while it influenced gram-positive strains mostly considering the only direct biofilm formation on the coatings. On the other hand, Zn coatings showed a more pronounced effect against the gram-positive *S. aureus* and *E. faecalis* strains considering both the planktonic and biofilm cells. These results are in line with several previous works that reported diversified bacterial responses to the metal toxicity according to the Gram staining categorization [39, 40]. Possible reasons for this different response to metal toxicity are the very different structures, thickness, and composition of the cell envelope/membrane [39, 41] as well as the distinct metal uptake and efflux systems that are present in two bacterial groups [42]. Electrostatic forces involving the negatively charged cell surface components (*i.e.*, lipopolysaccharides in gram-negative strains and teichoic and lipoteichoic acids in gram-positive strains) and the positively charged metals mediate the interaction between the bacterial cells and the toxic metals [43]. On the other hand, the thick cell wall composed of peptidoglycan (of gram-positive strains) and the hydrolytic enzymes in the periplasmic space (of gram-negative strains) can contrast the internalization of toxic metals [41]. In the literature, opposite data are reported regarding the resistance/sensitivity of these two Gram staining groups in the presence of zinc that was frequently tested in the form of ZnO nanoparticles (NPs) [44]. These works suggested that the thick peptidoglycan layer composing gram-positive bacterial cell wall could promote ZnO binding to the cell [45]. ZnO antibacterial activity could be associated with the production of reactive oxygen species (ROS), the loss of cellular integrity following contact between ZnO NPs and the cell wall, and the release of Zn^2+^ ions [45]. Zn^2+^ ions are inhibitors of ATP synthesis and oxidative phosphorylation in cells, causing membrane depolarization [46]. Similar to these previous studies, the antibacterial and antibiofilm activity of the IJD-derived zinc coatings we observed in our work could be due to a synergistic toxic effect of ZnO and of Zn^2+^ ions and/or to a first ZnO adsorption phase onto the cell surface with the subsequent intracellular release of Zn^2+^ ions over time. In this way, therefore, IJD-derived ZnO coatings can directly act on the bacterial attachment and biofilm formation and interact with planktonic cells suspended in the growth medium. Different morphology and roughness of the ZnO films, compared to Ag films, also play an important role, not only because they determine higher release of Zn but also because morphology can impact differently on bacterial cells adhesion.

While for both Ag and Zn the bacterial cell membrane/envelope represents one of the main targets of toxicity, additional toxic effects involve the inhibition of enzymatic processes and the production of reactive oxygen species (ROS) [47]. These additional toxicity targets could explain the differences we observed between the tested bacterial strains that belong to the same Gram staining group. For instance, the observation of a significant level of biofilm inhibition on the coated wells not only with the two gram-negative strains but also with *E. faecalis*, indicate that the two gram-positive strains differ in terms of their resistance against Ag coatings, being *S. aureus* the most resistant between the two gram-positive strains. Similarly, *P. aeruginosa* showed higher resistance to zinc as compared to *E. coli* considering both planktonic and biofilm cells. These resistance/sensitivity differences can be associated to genetic and molecular characteristics that are specific for each strain tested, independently of the general cell envelope organization shared by gram-positive and gram-negative strains. Proteomic studies identified several molecular targets of the silver toxicity in strains belonging to *P. aeruginosa*, *E. coli*, and *S. aureus*. These studies indicated that silver binds and inhibits the activity of intracellular proteins that are different in each strain and are primarily involved in the central metabolism, stress response, energetic metabolism, and DNA replication [34]. Transcriptomic analysis of a *S. aureus* MRSA strain exposed to ZnO-based NPs showed that while the genes involved in oxidative stress were differentially expressed only at low levels, the genes involved in uridine monophosphate (UMP) biosynthesis and carbohydrate degradation were significantly up-regulated. The association of UMP pathway with the anaerobic metabolism of *S. aureus* was suggested to be related to its higher sensitivity to ZnO NPs as compared to *E. coli* [48]. These data assess that bacteria-specific stress response is induced in the presence of silver and zinc that involves intracellular components; these mechanisms contribute to the interaction of each bacterial strain with the toxic metal implementing the various protective roles the cell membrane and cell wall [49, 50].

The intracellular localization of toxicity targets could also be partly related with the general lower values of adhesion inhibition we observed for the coatings (on the titanium alloys) as compared to the biofilm inhibition (on CBD). Indeed, during the bacterial adhesion stage (the first biofilm formation step), electrostatic forces are mostly involved which mediate the bacterial cell envelop contact with the coating surface [51]. As the biofilm formation proceeds, the bacterial interaction with the coating takes place and the metal ions released increase. The cellular internalization of the metal thus occurs that is toxic for intracellular targets leading to cell death, as we could also observe through SEM on the coatings on the titanium alloys.

The use of CBD system allowed us to define the antibiofilm activity associated with both the direct cellular contact with the coating and the soluble metal ions released by the coatings. Indeed, the toxicity of the soluble metal ions could be detected by analysing the effect on the planktonic cells and on the biofilms formed on the uncoated CBD peg. The direct effect of the bacterial contact with the metal coating on biofilm formation could be analysed by evaluating the biofilm formed on the coated CBD well. Our results showed that, for all the bacterial strains tested, the major antibiofilm activity of the Ag coatings was exerted on the biofilms formed directly on the coatings. The silver ions released from the Ag coatings mostly inhibited the planktonic cells growth and only to a lower extent the biofilm formation on the uncoated peg (Fig. 5). This can be related with previous results that showed Ag NPs to be mostly active against bacteria through a contact killing mechanism [52]. However, in the case of Zn coating activity against gram-positive strains, the ions released showed a strong effect against biofilms formed on uncoated pegs. In some cases, this toxic effect was even higher than that observed on the biofilms directly formed on the coating. This could be associated with the high amounts of metal ions released by the Zn coatings (as compared to the Ag coatings), the high toxicity of zinc against gram-positive strains, and the effect of the irregularity and roughness of the zinc coating on these strains’ adhesion. The antibiofilm activity of the Ag and Zn coatings detected on the CBD system was further analysed by considering the anti-adhesion properties of these coatings on titanium alloys that mimic real materials used for orthopaedic implants. The anti-adhesion experiments confirmed the higher activity of Ag coatings against gram-negative strains and the strong activity of Zn coatings against gram-positive strains. The inhibition values were generally lower than those detected from antibiofilm tests probably due to the different experimental conditions used (medium volume and initial bacterial concentration); furthermore, the extension of the metal coated area was significantly lower in the case of titanium alloys that had a coated face while the surrounding parts were not coated, being therefore exposed to possible bacterial adhesion. On the other hand, the microscopy analysis of the biofilm formation on the coated side of the titanium alloys completely supported the inhibitory data obtained from CBD. The observations showed modified cellular morphology related with cell breakage, damage, and/or biofilm eradication (like in the case of *S. aureus* on Ag coating).

Both Ag and Zn coatings showed suitable extent and duration of the ion release. Indeed, metal release is still increasing after 7 days in medium, indicating that both coatings are suitable for prevention of early infections (*i.e.*, those occurring < 2 weeks after implantation [53, 54]), that constitute the large majority of orthopaedic infections. To study the capability to address late infections (> 10 weeks) and the overall duration of the antibacterial release, new protocols are needed for accelerated ageing of the films, which are currently under investigation. Finally, no cytotoxicity was assessed for any of the two films.

These results extend and validate the utilization and possible application of these coatings on real implementation devices for bacterial infection prevention.

## Conclusions

One of the most promising strategies to solve the problem of bacterial infections related to biomedical devices implant is the employment of metal coatings with antimicrobial properties. In this work we have demonstrated that the combination of Ionized Jet Deposition with Calgary Biofilm Device is a high-throughput, rapid, and reproducible tool for the screening of new generation metal coatings with possible applications in biomedical field. IJD-deposited films covering CBD were deposited at three different concentrations and were composed of nanosized spherical aggregates of metallic silver or zinc oxide with a fine and highly rough surface topography. The study of both antibacterial (against suspended cells) and antibiofilm (against biofilm cells) properties showed the high efficacy of Ag and Zn coatings against gram-negative and gram-positive strains, respectively. The efficacy of the antibacterial coatings was defined against two types of biofilms, *i.e.*, the biofilm adhering and growing directly on the coating and the biofilm developing on a substrate that is not coated (the CBD peg). Our work demonstrates that the metal coatings were most efficient against biofilms directly growing on the coatings; however, we also detected a significant level of efficacy against the biofilms on uncoated pegs of the CBD. The antibiofilm properties were, however, strictly associated with the metal and bacterial strain under analysis. This is an important result to consider, as in a prosthetic implant system both these types of biofilm can develop. Indeed, the coating is on the prosthesis surrounded by possible alternative substrates for the biofilm that are not coated (*i.e.*, the proximal bones and tissues). In addition, our results show that the CBD is a powerful tool, that permits to take into consideration both the metal ions release and the surface topography of the films, which makes it particularly suitable for the study of the antibacterial efficacy of nanostructured materials. The results obtained with the CBD were validated with coatings on titanium alloys and extended by also considering the anti-adhesion properties and the cytotoxicity, for application to orthopaedic prostheses. The coatings were effective and non-cytotoxic, hence they appear promising for application to both surfaces and implantable devices. For zinc, for which some reduction was found in L929 viability, possible application in combination with a bioactive ceramic, that can promote host cells behavior, is foreseen. Finally, the results will need to be validated in vivo. In view of upcoming application in orthopaedics, these evaluations would be useful for the development of materials with pleiotropic antimicrobial mechanisms.

## Methods

### Ionized Jet Deposition applied onto Calgary Biofilm Devices and titanium alloys

All coatings were obtained by Ionized Jet Deposition, starting from commercial metallic silver and zinc targets (Kurt J. Lesker, PA, USA and EVOCHEM Advanced Materials, respectively). In IJD, a solid target material, either a metal or a ceramic (here, metallic silver and zinc, respectively), is ablated by a pulsed electron beam. The interaction with the beam causes the ionization of the target, and the ionized material is accelerated towards the substrate where it grows in the form of a nanostructured thin film.

Here, for the deposition, the targets were mounted on a rotating holder inside the deposition chamber ablated by the electron gun (fast pulse—100 ns of high energy—10 J electrons with high-density power). The first 5 min of deposition were performed on a shutter to avoid deposition of surface impurities deriving from the target very surface. The chamber was initially evacuated down to a base pressure of 1.0 × 10^–7^ mbar by a turbomolecular pump (EXT255H, Edwards, Crawley, UK) and then pressure was raised by a controlled flow of oxygen (purity level = 99.999%) up to 3 × 10^–4^ mbar.

Deposition parameters were selected based on past results. More in detail, target-substrate distance, electron beam frequency, and working voltage were optimized to ensure uniformity of deposition and adjusted to 8 cm, 22 Hz, and 7 kV, respectively. Deposition time was fixed and set to 30 min, while different coatings characteristics and ion release were obtained by comparing different areas of the substrate. Indeed, the exact characteristics (thickness, surface roughness, aggregates geometry) depend on the distance from the plasma plume and its angle of incidence with the substrate.

Calgary Biofilm Devices were used as deposition substrates. As a results of the deposition on the CBD, three areas were selected in the Calgary, summarized in Fig. 1, having the same distance and angle with respect to the plasma plume and hence the same characteristics of the coating. In the CBD, only the wells showing complete coatings and high homogeneity were selected, while the others were discarded. Indeed, in IJD, the characteristics of the coatings depend on the distance and the angle of the plasma plume. As a consequence, the areas that are more distant from the plasma plume and have lower angles of incidence suffer relevant shadowing and incomplete coverage, both hampering a reliable evaluation of antimicrobial activity. All the compositional, morphological, and microbiological analyses performed in this work involved the three different areas selected on the CBD.

Then, to validate the approach, micro-rough medical grade titanium alloy disks (Grade 23 Titanium 6Al-4 V ELI alloy, 5 mm of thickness, 5/10 mm of diameter, Citieffe S.r.l.) were used as substrates for the deposition, after ultrasonic cleaning in isopropyl alcohol and water. A surface roughness (Ra) of 5 μm was specifically selected as representative of that of orthopaedic implants. Cylinders were placed in the deposition chamber, so as to correspond to zone “M” in the CBD.

### Composition and morphological characterization of the coatings

The coatings morphological characteristics were analysed by evaluating their morphology and surface geometry by Field Emission Gun Scanning Electron Microscopy (FEG-SEM) and Atomic Force Microscope (AFM). To avoid cutting of the CBD, plastic coverslips of the same material and geometry of the wells were inserted in the different wells of the CBD and extracted for microscopy analyses. Coatings uniformity and surface texture associated with each CBD area were assessed by FEG-SEM (Tescan Mira3, CZ, working distance = 10 mm, voltage = 5 kV).

For samples topography and thickness a Multimode VIII AFM equipped with a Nanoscope V controller (Bruker, USA) was used. The AFM was operated in the ScanAsyst Imaging mode using a NT-MDT cantilever (NSG10, nominal spring constant 3.1 N/m). Based on the acquired images, minimum and maximum diameters of the metal aggregates and the clusters were determined by ImageJ. Surface roughness was calculated as root mean square (RMS) by Gwyddion after performing a plane subtraction 0 order line-flattening [24]. Thickness was measured by optical profilometer after scratching the samples.

Films composition was assessed by XRD, using a Malvern PANalytical Empyrean series III instrument (40 kV and 30 mA, 2θ range = 20–80, step size = 0.01, time per step = 30 s). XRD was performed on the thicker coatings (H), that were deposited on 2 × 2 mm^2^ glass slides, so that no bands deriv-420ing from the substrate could conceal those of the coating.

Silver and zinc ions concentration in the wells were measured by Inductively Coupled Plasma (ICP). In particular, the metal-coated CBD wells were filled with 150 µL Luria–Bertani (LB) medium (NaCl 1% w/v, tryptone 1% w/v, yeast extract 0.5% w/v) that was kept in the wells for 48 h, before being collected for ICP analysis. The media from all the wells belonging to the same CBD area (minimum 4 wells) were collected and pooled together. The data acquisition was carried out in triplicates, using the media from wells of three different CBD separately coated and analysed. To determine the concentration of Ag and Zn ions in the medium, the standards and samples were first diluted in a nitric acid solution with a final concentration of 4% (v/v) in ASTM type IV deionized water (Millipore MilliQ IQ7000). Calibration curves were obtained using standard solutions. To obtain the curves, a multi-element solution IV-ICPMS-71A (Inorganic Ventures) was used and progressively diluted. An Yttrium solution (100 ppb) was used as internal standard (isotope 89Y, obtained by dilution of a 1000 ppm solution, VWR). Data were collected by using an ICP-MS instrument (XSeriesII, ThermoFisher Scientific) with the isotopes 66Zn e 107Ag.

### Preparation of the bacterial cultures for the antibacterial and antibiofilm assays

Four pathogenic bacteria were tested, *i.e.*, the gram-negative strains *Escherichia coli* ATCC 8739 and *Pseudomonas aeruginosa* PAO1, and the gram-positive strains *Staphylococcus aureus* ATCC 6538P, and *Enterococcus faecalis* DP1122. All cultures were conducted using LB medium, to which agar (1.5% w/v) was added to generate solid LB plates. The study was carried out by inoculating a single colony of each strain (grown on agar plate for 24 h) in 50 mL tubes with 5 mL of LB liquid medium. Cultures were grown overnight at 37 °C under agitation at 150 rpm and then diluted to reach specific optical density values measured at 600 nm (OD_600_).

### Evaluation of bacterial planktonic and biofilm cell growth in the Calgary Biofilm Device

The cultures grown overnight (o/n) were diluted to reach an optical density of 0.03 measured at 600 nm (OD_600_). From these bacterial suspensions, a volume of 150 μL was transferred to each CBD well. The CBD plates were then incubated at 37 °C with gentle shaking at 100 rpm for 48 h in a humid environment to avoid desiccation. After incubation, the bacterial suspension was removed from each well and the OD_600_ was measured to evaluate the growth of planktonic cells. Biofilms formed on the wells and pegs were quantified through crystal violet (CV) staining.

After bacterial growth, the CBD wells and pegs were washed twice with saline solution (NaCl 0.85% w/v) and then fixed with 99% ethanol (v/v) for 10 min. Afterwards, wells and pegs were stained with an aqueous solution of CV (0.2% w/v) to stain the adhered cells and the biofilms by incubating the supports in the CV solution for 10 min at room temperature. The unbound dye was washed away with sterilized water. Lastly, 200 μL of an aqueous solution of acetic acid (33% v/v) was added to solubilize the stained biofilms and the optical density at 595 nm (OD_595_) was measured for biofilm quantification. Controls were performed on CBD under the same growth conditions using wells without metal deposition. The background staining was corrected by subtracting the mean value for CV bound to negative controls (CBD wells with the growth medium but without the bacterial inoculum).

### Evaluation of bacterial biofilm formation on Ag- and Zn-coated titanium alloys

For the anti-adhesion and antibiofilm assays, the bacterial suspensions grown o/n were diluted to reach OD_600_ of 0.2 and 0.03, respectively. Then, 1 mL of the diluted suspensions was transferred into each well of 24-wells microplates. Sterile alloys coated with Ag, with Zn, and without coating (as control) were inserted with the coating facing up in the bacterial suspension. The microplates were incubated for 4 h (for anti-adhesion assay) or 48 h (for antibiofilm assay) at 37 °C with gentle shaking (90 rpm). The alloys were then removed from the cultures to quantify the adhered cells through CV staining and to visualize the biofilm through SEM.

### Scanning Electron Microscopy of bacterial biofilms on Ag- and Zn-coated titanium alloys

The biofilms of the four bacterial strains were observed through SEM analysis on titanium alloys coated with silver, zinc, and without coating (as control experiment). Biofilms were produced by inoculating 700 μL of a bacterial suspension with OD_600_ = 0.03 in 48-multiwells plates with the titanium alloys and by incubating the plates at 37 °C for 48 h with gentle shaking. The titanium alloys were washed twice in NaCl 0.85% w/v, fixed in PBS 0.1 M pH 7.2 with glutaraldehyde 2.5% for 2 h, washed in PBS 0.1 M pH 7.2 for 10 min, and air-dried.

### Ion release from Ag- and Zn-coated titanium alloys

Release of Ag and Zn from the coatings was measured in DMEM + 2 mM L-glut + 10%PBS + 1%es. The supernatant liquids were removed from the wells at increasing times up to 7 days and metal content was analyzed by means of Agilent 4210 (Agilent, Santa Clara, CA, USA) Molecular Plasma-Atomic Emission Spectroscopy (MP-AES). Silver lines at 338.289 nm and Zinc line at 213.857 nm were used. The calibration lines were made with 4 calibration standards (Ag: 1, 6, 10, 15 mg/L; Zn: 2, 40, 100, 140 mg/L), prepared by dilution of 1000 mg/L silver or zinc standard solutions in 0.5 M HNO_3_. Results from this analysis represent the mean value of three different determinations on different samples.

### Cell cultures

Murine L929 fibroblasts cell line were purchased from the American Type Culture Collection (ATCC, Manassas, VA, USA). Cells were cultured in Dulbecco’s Modified Eagle’s Medium (DMEM, Life Technologies, UK) with 10% fetal bovine serum (FBS, Sigma-Aldrich, Milan, Italy), 1% penicillin/streptomycin, and 2 mM L-glutamine (Life Technologies). Cells were maintained at 37° C in a humidified 5% CO_2_ atmosphere.

### Cell viability

The Ag and Zn cytotoxicity was tested using eluates from the two coatings, selecting the concentration corresponding to the highest ion release (*i.e.*, 7 days). Zn/Ag coated plates were kept in medium (D-MEM, Gibco) at 37° C in a humidified 5% CO_2_ atmosphere on a plate-shaker (60 rpm). After 7 days the eluates were collected.

The cytotoxicity was evaluated by methylthiazolydiphenyl-tetrazolium bromide (MTT) assay. The assay is a colorimetric test based on the ability of viable cells to convert MTT solution in water-insoluble MTT formazan by mitochondrial dehydrogenases.

Briefly, 2x10^3^ cells/well were plated in flat-bottomed 96 microplate wells (Costar, Cambridge, MA) in complete D-MEM. After cell adhesion, Ag and Zn eluates were added to culture medium and kept in contact with the cells for 24 h. Cells treated with medium conditioned with Ti alloy plates without Ag/Zn coatings were used as controls. After 24 h, cells were exposed to 5 mg/mL of MTT in complete medium for 4 h at 37 °C, washed with PBS, and 100 μL DMSO was added to each well to dissolve the MTT-formazan crystals. The absorbance was measured at 570 nm by a plate reader spectrophotometer (Tecan Infinite F200pro, Mannedorf, Switzerland). Results were recorded as optical density units (OD_570_) and averaged after blank subtraction. Data were expressed as percentage between OD measured in samples exposed to eluate and OD measured in negative control. The experiment was repeated two times in quadruplicate.

### Data analysis

The growth inhibition (% inhibition in Fig. 5) was calculated as follows: % inhibition = (1 – T/C) × 100, where T and C are cell density (measured as OD_600_ for planktonic and OD_595_ for biofilm) in the target experimental samples (inoculated CBD wells with coatings) and control samples (inoculated CBD wells without coatings), respectively. All the microbiological and cytotoxicity results are expressed as average ± standard deviation (n > 3). Statistical significance was determined through a one-way Anova test in Graphpad Prism. Differences were considered significant when *p* < 0.05.

## Supplementary Information

**Additional file 1: Figure S1.**

**Additional file 2: Table S1**.

## Data Availability

All data generated or analysed during this study are included in this published article.
