# Supplementary figures and images for: Customized biofilm device for antibiofilm and antibacterial screening of newly developed nanostructured silver and zinc coatings
Source: J Biol Eng. 2023 Mar 6;17:18. doi: 10.1186/s13036-023-00326-y (PMC9987098; doi:10.1186/s13036-023-00326-y)

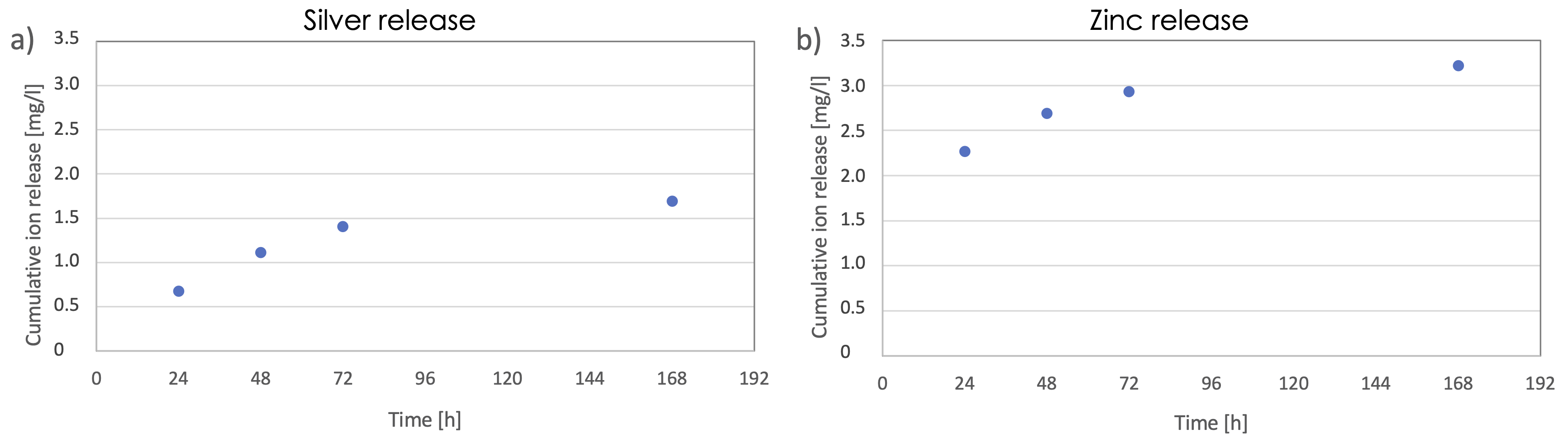

Supplement: Supplementary file 1 — Additional file 1: Figure S1. [file 13036_2023_326_MOESM1_ESM.tiff]
