# Supplementary material for: Customized biofilm device for antibiofilm and antibacterial screening of newly developed nanostructured silver and zinc coatings
Source: J Biol Eng. 2023 Mar 6;17:18. doi: 10.1186/s13036-023-00326-y (PMC9987098; doi:10.1186/s13036-023-00326-y)
Supplement: Supplementary file 2 — Additional file 2: Table S1. [file 13036_2023_326_MOESM2_ESM.docx]

**Table S1**. Absorbance values (OD_595_) of crystal violet associated with the amount of biofilm on wells and pegs without metal coating (*i.e.*, control experiments in Figure 5).

| Bacterial strain | Biofilm on wells | | Biofilm on pegs | |
| --- | --- | --- | --- | --- |
|  | Mean value OD_595_ | Standard deviation | Mean value OD_595_ | Standard deviation |
| *E. coli* | 1.210 | 0.082 | 0.841 | 0.099 |
| *P. aeruginosa* | 2.839 | 0.194 | 1.881 | 0.103 |
| *S. aureus* | 2.768 | 0.154 | 1.125 | 0.056 |
| *E. faecalis* | 0.989 | 0.069 | 0.657 | 0.085 |
